# Convergence between a mosquito-eating predator's natural diet and its prey-choice behaviour

**DOI:** 10.1098/rsos.160584

**Published:** 2016-12-07

**Authors:** Robert R. Jackson, Chan Deng, Fiona R. Cross

**Affiliations:** 1 School of Biological Sciences, University of Canterbury, Private Bag 4800, Christchurch 8140, New Zealand; 2 International Centre of Insect Physiology and Ecology, Thomas Odhiambo Campus, PO Box 30, Mbita Point, Kenya

**Keywords:** specialization, preference, stenophagy, Salticidae, *Evarcha culicivora*, *Anopheles gambiae*

## Abstract

On the basis of 1115 records of *Evarcha culicivora* feeding in the field, we can characterize this East African jumping spider (Salticidae) as being distinctively stenophagic. We can also, on the basis of laboratory prey-choice experiments, characterize *E*. *culicivora* as having a specialized prey-classification system and a hierarchy of innate preferences for various categories of mosquitoes and other arthropods. Prey from the field belonged to 10 arthropod orders, but 94.5% of the prey records were dipterans. Mosquitoes were the dominant prey (80.2% of the records), with the majority (82.9%) of the mosquitoes being females, and thereafter midges were the most common prey (9.2% of the records). Preference profiles that were determined from experiments showed strong convergence with natural diet in some, but not all, instances. In experiments, *E*. *culicivora* adults appeared to distinguish between six prey categories and juveniles between seven, with blood-carrying anopheline female mosquitoes being ranked highest in preference. For adults, this was followed by blood-carrying culicine female mosquitoes and then anopheline female mosquitoes not carrying blood, but these two preferences were reversed for juveniles. Moreover, for juveniles, but not for adults, anopheline male mosquitoes seem to be a distinct prey category ranked in preference after blood-carrying culicine females and, for both adults and juveniles, preference for midges is evident when the alternatives are not mosquitoes. These findings illustrate the importance of going beyond simply specifying preferred prey categories when characterizing predators as ‘specialized’ and a need to make clear conceptual distinctions between a predator's natural diet, the prey categories that are relevant to the predator, and the predator's prey-choicebehaviour.

## 1. Introduction

Making a clear distinction between preference and natural diet is important when discussing predatory specialization [1] because, although natural diet is simply what a predator eats in the field, preference is an inherent product of a predator's perceptual processes, decision-making capacities and motivation. A combination of laboratory experiments and field sampling is necessary for determining the extent to which preferences and natural diet converge but, in research on many predators, this combination is often unrealistic. However, *Evarcha culicivora*, the predator we consider here, is an exception because a long-term research programme on this jumping spider (family Salticidae) from East Africa has allowed for large datasets from the field pertaining to natural diet and large datasets from laboratory prey-choice experiments pertaining to preferences.

Spiders are usually characterized as being ‘generalist predators’ (e.g. [2–4]) but often, when reading the literature on spiders and other predators, it is difficult to discern whether ‘generalist’ refers to euryphagy (i.e. inclusion of a wide range of prey in the predator's natural diet), indiscriminate feeding (i.e. the absence of pronounced prey-choice behaviour) or some combination of the two (see [1]). It is particularly misleading when the expression ‘generalist predator’ is used for characterizing the prey-choice behaviour of salticid spiders. The majority of well-designed experimental studies on salticids have revealed distinct preferences, the most notable examples coming from salticids that target ants as preferred prey [5] and from other salticids that target spiders as preferred prey [6]. Even if the term ‘generalist’ is being used when the intended term is ‘euryphagy’, it is premature to conclude that salticids as a group can be characterized this way because tabulated data pertaining to natural diet are scarce for this large spider family of almost 6000 described species [7].

That *E*. *culicivora* is a salticid has been an important factor in our research because salticids have unique, complex eyes and an ability to see prey in remarkably fine detail [8,9], these being characteristics that contribute to these predators being unusually cooperative subjects in prey-choice experiments. For example, even when distant from its prey, a salticid can initiate distinctive predatory behaviour which a scientist may record and use as evidence of a salticid's decisions [10]. Moreover, in research on salticids, prey-choice experiments can be designed in ways that avoid the risk of experimental outcomes being influenced by uncontrolled prey behaviour. Many salticids [10], including *E*. *culicivora* (table 1), are known to respond to lures (dead prey mounted in life-like posture on cork discs) or even to virtual prey generated by computer animation software (e.g. [14]), with these responses corresponding well to how they respond to living, active prey [1].

**Table 1.** Summary of experiments demonstrating that *Evarcha culicivora* expresses preference for indirect blood meals. Unless otherwise stated, ‘adult’ test spiders were males and females. For each pair of prey, significantly more test spiders chose prey 1 (blood-carrying female mosquito) instead of prey 2 (not carrying blood). *Ae*.: *Aedes*. *An*.: *Anopheles*. *Cx*.: *Culex*.

prey 1 (blood)	prey 2 (no blood)	test spiders	stimuli	study
*Ae*. *aegypti*	*Ae*. *aegypti* female	adults and juveniles	stationary lures	[11]
*Ae*. *aegypti*	*Ae*. *aegypti* male	adults and juveniles	stationary lures	[11]
*Cx*. *quinquefasciatus*	*Cx*. *quinquefasciatus* female	adults and juveniles	stationary lures	[11]
*Cx*. *quinquefasciatus*	*Cx*. *quinquefasciatus* male	adults and juveniles	stationary lures	[11]
*An*. *funestus*	*An*. *funestus* male	adults and juveniles	stationary lures	[11]
*An*. *gambiae*	*An*. *gambiae* female	adults and juveniles	stationary lures	[11]
*An*. *gambiae*	*An*. *gambiae* male	adults and juveniles	stationary lures	[11]
*An*. *gambiae*	*An*. *gambiae* male	adult females	moving lures	[12]
*An*. *gambiae*	*An*. *gambiae* male	adult females	living prey	[13]
*An*. *gambiae*	ghost midge: *Chaoborus* sp.	adults	stationary lures	[11]
*An*. *gambiae*	chironomid midge: *Ablabesmyia nilotica*	adults	stationary lures	[11]
*An*. *gambiae*	chironomid midge: *Chironomus imicola*	adults	stationary lures	[11]
*An*. *gambiae*	chironomid midge: *Clinotanypus claripennis*	adults	stationary lures	[11]
*An*. *gambiae*	chironomid midge: *Clinotanypus claripennis*	adult females and juveniles	moving lures	[12]
*An*. *gambiae*	chironomid midge: *Clinotanypus claripennis*	adult females	living prey	[13]
*An*. *gambiae*	chironomid midge: *Conochironomus acutistus*	adults	stationary lures	[11]
*An*. *gambiae*	chironomid midge: *Nilodorum brevibucca*	adults and juveniles	stationary lures	[11]
*An*. *gambiae*	aphid: *Brevicoryne brassicae*	adults and juveniles	stationary lures	[11]
*An*. *gambiae*	caterpillar *Chilo parttelus*	adults	stationary lures	[11]
*An*. *gambiae*	fruit fly: *Ceratitis capitata*	adults	stationary lures	[11]
*An*. *gambiae*	nephilid spider: *Nephilengys*	adults and juveniles	stationary lures	[11]
*An*. *gambiae*	oecobiid spider: *Oecobius amboseli*	adults	stationary lures	[11]

*Evarcha culicivora*'s predatory strategy is strikingly unusual. This spider feeds indirectly on vertebrate blood by actively choosing blood-carrying female mosquitoes as its preferred prey [11] and, by actively choosing *Anopheles* as its preferred mosquitoes [15], it singles out the particular mosquito genus to which all human malaria vectors belong [16]. Vision is not the only sensory modality that matters to *E*. *culicivora*, with olfaction in particular having various [1], and sometimes surprising (e.g. [17]), roles in this species' biology. However, vision-based prey-choice behaviour by *E*. *culicivora* has been the most thoroughly investigated and, regardless of whether living prey, lures or virtual prey were used, all earlier studies have confirmed this salticid's distinctive preference for blood-carrying mosquitoes (table 1).

In the most comprehensive study of *E*. *culicivora*'s preferences to date [12], lures were made from a non-biting midge species, *Clinotanypus claripennis* (Chironomidae), and from both sexes of two mosquito species, *Anopheles gambiae s*.*s*. and *Culex quiquefasciatus*. For both mosquito species, a distinction was made between females that carried blood and females that did not carry blood. The methods and findings from this earlier study are strongly connected to our objectives in this study.

The experimental methods used by Nelson & Jackson [12] were designed for ascertaining the strengths of *E*. *culicivora*'s different preferences. Three testing protocols (simultaneous, alternate-day and alternative-prey) were used (table 2) and, for each protocol, individuals were subjected to pre-trial fasts of different durations. In simultaneous testing, *E*. *culicivora* could choose one of two lures; both were present at the same time, one lure being made from one kind of prey and the other being made from another kind of prey. In alternate-day testing, *E*. *culicivora* was shown a single lure of one type on one day and a single lure of another type on the next day (i.e. each test spider was used in a pair of tests), with only those test pairs in which *E*. *culicivora* chose one prey, but not the other, being used as data for determining preference. In alternative-prey testing, *E*. *culicivora* was again shown a lure of one type on one day and a lure of another type on the next day. However, in these tests, the spider was shown a lure of one type while feeding on an individual of the other prey type, with a ‘choice’ being recorded only if the spider dropped the prey it was eating when approaching the lure. The prey type that the spider was eating on the first day was used as a lure on the second day.

**Table 2.** Preference strengths of *Evarcha culicivora* with respect to specific pairs of prey. Determined from data of Nelson & Jackson [12]. Mosquitoes used: *Anopheles gambiae s*.*s*. and *Culex quinquefasciatus* (shortened to *Anopheles* and *Culex*). Female (f) and male (m) mosquitoes used. Midge (Chironomidae): *Clinotanypus claripenni*. Strong preference for prey 1: significantly more test spiders chose prey 1 than chose prey 2 after a 14-day pre-trial fast. Medium preference for prey 1: significantly more test spiders chose prey 1 than chose prey 2 after a 7-day fast, but not after longer fast. Weak preference for prey 1: no significant choice after longer fasts, but significantly more test spiders chose prey 1 than chose prey 2 after a 1-day fast. One instance of significantly more test spiders choosing prey 2 than prey 1. Nil preference: no significant choice detected after 1-day, 7-day or 14-day fasts.

		adult test spider			juvenile test spider		
prey 1	prey 2	simultaneous presentation	alternate day	alternate prey	simultaneous presentation	alternate day	alternate prey
blood *Anopheles* (f)	blood *Culex* (f)	weak^a^	weak	nil	medium^a^	weak	nil
	no-blood *Anopheles* (f)	strong	medium	weak	medium	medium	nil
	no-blood *Culex* (f)	strong	medium	weak	medium	medium	nil
	*Anopheles* (m)	strong	medium	weak	medium	medium	nil
	*Culex* (m)	—	—	—	—	—	—
	midge	strong	medium	nil	medium	medium	nil
blood *Culex* (f)	no-blood *Anopheles* (f)	strong	medium	weak	weak^b^	nil	nil
	no-blood *Culex* (f)	strong	medium	weak	medium	weak	nil
	*Anopheles* (m)	—	—	—	—	—	—
	*Culex* (m)	strong	medium	weak	medium	weak	nil
	midge	strong	medium	weak	medium	weak	nil
no-blood *Anopheles* (f)	no-blood *Culex* (f)	weak^c^	weak	nil	medium^c^	medium	nil
	*Anopheles* (m)	weak	weak	nil	medium	weak	nil
	*Culex* (m)	weak	weak	nil	medium	weak	nil
	midge	weak^c^	weak	nil	medium^c^	weak	nil
no-blood *Culex* (f)	*Anopheles* (m)	nil	nil	nil	nil	nil	nil
	*Culex* (m)	nil	nil	nil	nil	nil	nil
	midge	nil	nil	nil	nil	nil	nil
*Anopheles* (m)	*Culex* (m)	nil	nil	nil	medium	weak	nil
	midge	nil	nil	nil	weak	nil	nil
*Culex* (m)	midge	nil	nil	nil	nil	nil	nil

^a^Corresponds to preference found by Nelson & Jackson [15] when using lures and when using virtual prey.

^b^In this instance, preference for prey 2 (i.e. juvenile's preference for *Anopheles*) over-rides preference for blood.

^c^Corresponds to preference found by Nelson & Jackson [15] when using lures.

Irrespective of whether test spiders were adults or juveniles, Nelson & Jackson [12] found a primary preference for blood-carrying female mosquitoes, but preference for blood meals was expressed more strongly by adults than by juveniles. In addition, adults and juveniles chose female mosquitoes in preference to male mosquitoes (also see [18]) and anopheline mosquitoes in preference to culicine mosquitoes (also see [14,15,19]), but the preference expressed by juveniles for anophelines was stronger than the preference expressed by adults for anophelines (table 2). One of the most important implications of these findings is that, when characterizing predators as ‘specialized’, it is important to consider preference profiles instead of simply specifying a preferred prey category.

Yet our understanding of *E*. *culicivora*'s preference profile has remained incomplete. In previous research, only a limited number of non-mosquito prey species were used and at least one of the prey individuals was always a mosquito (tables 1 and 2). For example, the only non-mosquito prey species used by Nelson & Jackson [12] and by Jackson & Nelson [13] was *C*. *claripennis*, a chironomid midge, whereas a different chironomid midge, *Nilodorum brevibucca*, was the only non-mosquito prey species that Nelson & Jackson [15] used when making lures. Experiments using virtual prey by Nelson & Jackson [15] and by Dolev & Nelson [14,19] have been based primarily on the way the resting postures of anophelines and culicines differ [20], although Dolev & Nelson [14,19] also used virtual house flies (*Musca domestica*). Jackson *et al*. [11] used a wider range of non-mosquito prey (aphids, caterpillars, fruit flies and spiders, as well as six midge species) but the lures were always stationary and presented simultaneously and, in that study, the pre-trial fast was always 7 days (table 1).

One of our goals has been to extend our understanding of the prey-choice behaviour of *E*. *culicivora*. Here, we use a wider range of non-mosquito species and also, for the first time, we include experiments in which non-mosquito species are paired with other non-mosquito species. Being interested in specifically innate preferences, we endeavoured to standardize test spiders' prior experience with prey. Another goal has been to link our prey-choice experiments more closely than has been the case in the past to an understanding of this predator's natural diet. For this goal, we were guided when selecting the prey to use in our experiments by having more than 1000 records of prey eaten by *E*. *culicivora* in the field. This large sample of prey from the field, combined with a wide range of experiments designed specifically for gaining insight into how *E*. *culicivora* innately categorizes prey, has given us an unprecedented opportunity to determine the extent of convergence between a predator's natural diet and its innatepreferences.

## 2. Material and methods

### 2.1. Prey records from the field

Our field site was the town of Mbita Point in western Kenya, including the Thomas Odhiambo Campus of the International Centre of Insect Physiology and Ecology (icipe; elevation 1200 m.a.s.l., latitude 0°25′S–0°30′S, longitude 34°10′ E). All required approvals and permits for carrying out our research were included in visiting scientist contracts with icipe (R.R.J. since 1994; F.R.C. since 2006) and, for C.D., a student internship under icipe's Dissertation Research Internship Programme. We accumulated records of prey in the field by adopting a simple, informal method. When we and other personnel from our Mbita Point laboratory saw an individual of *E*. *culicivora* (the ‘predator’) in the field that was the act of feeding, we put this predator, along with its prey, in a plastic vial and then we separated the predator from its prey by shaking the vial or, by using a soft brush, prodding the predator until it released the prey. In the laboratory, we identified the prey and also recorded whether the predator was an adult female, an adult male or a juvenile. We used forceps to press on the abdomen of any female mosquito taken from *E*. *culicivora* and, whenever red or reddish brown fluid was notable, we recorded that the female mosquito was carrying blood. Besides using our new prey records from 2002 to 2015, we include the 202 records from work in 1994, 1995, 1997, 1998 and 2001 [21] which were collected using the same procedure.

### 2.2. Laboratory procedures

On the whole, we used apparatus, basic experimental procedures and basic laboratory rearing protocols that have been described elsewhere in greater detail [12]. However, the specific prey pairings we used here and the prey pairings used by Nelson & Jackson [12] were different and, although some of the particular pairings of prey types had been used in the first study of *E*. *culicivora*'s prey-choice behaviour [11], the apparatus and testing procedures used here, and by Nelson & Jackson [12], were considerably different (table 1).

Our test spiders were adult females (4.5–5.0 mm in body length) as well as mid-size juveniles (3.0 mm). We decided not to use adult males because, although no male–female differences in *E*. *culicivora*'s preference were detected in the previous research (table 1), salticid males tend to be less responsive than salticid females to prey [22]. We adopted 3.0 mm as the standard body length for juvenile test spiders because, in earlier experiments [12] (table 2), juvenile–adult divergence in preference was evident only when juveniles were 3.5 mm or less in body length. We decided not to use juveniles that were smaller than 3.0 mm because we wanted to avoid a large juvenile–adult size disparity. All test spiders came from laboratory cultures and the body length of each test spider was accurate to the nearest 0.5 mm.

For standardization, all adult test spiders matured in the laboratory two to three weeks before being used and none had mated. The standard rearing diet in the laboratory after spiders emerged from eggsacs until time used in experiments was blood-carrying female mosquitoes (*Anopheles gambiae sensu stricto*) and midges (*Nilodorum brevibucca*), with both prey types being provided ad libitum every Monday, Wednesday and Friday. Owing to our goal being to ascertain *E*. *culicivora*'s innate preference profile, we used test spiders that had been on this standard diet in most of the prey-choice experiments. The only exception pertained to determining whether experience with a non-preferred prey might alter test-spider preference. For this, some of the test spiders in one subset of prey-choice experiments in the ‘complete series’ (see below) were on an alternative rearing diet (‘spider-only’) in which the only food received was oecobiid spiders after emergence from eggsacs until time used in experiments. Findings from other experiments in our present study showed no evidence of test spiders on the standard diet expressing any preference for oecobiid spiders. To minimize rearing time, we only used juveniles as test spiders for the spider-only diet.

#### 2.2.1. Lures

In all experiments, we used lures instead of living prey (table 3), with the body length of each lure being accurate to the nearest 0.5 mm. No lure was used in more than one trial. The mosquitoes that we used for making lures were *Anopheles gambiae sensu stricto* and *Culex quinquefasciatus* (henceforth referred to simply as *Anopheles* and *Culex*). For both mosquito species, there were three types (males, blood female and no-blood female), making a total of six mosquito types used in experiments. ‘Blood females’ received a blood meal 4 h before being fed to the spiders that were maintained on the standard diet or before being used for making lures. ‘No-blood females’ received no blood meals, but all mosquito types had unrestricted access to glucose (6% solution).

**Table 3.** Arthropods used as lures in this study.

common name	genus, species	family	order	body length (mm)
assassin bug^b,d^	*Nagusta*	Reduviidae	Hemiptera	4.0
aphid^b,c^	*Brevicoryne brassicae*	Aphidae	Hemiptera	3.0
barklouse^b^^,^^d^	unidentified	unidentified	Psocoptera	3.0
brown rice hopper^a^^,^^d^	*Nilaparvuta lugens*	Delphacidae	Hemiptera	3.0
caterpillar^a^^,^^c^	*Chilo parttelus*	Crambidae	Lepidoptera	4.5
chironomid midge^b^^,^^e^	*Ablabesmyia nilotica*	Chironomidae	Diptera	4.0
chironomid midge^b^^,^^e^	*Chironomus imicola*	Chironomidae	Diptera	5.0
chironomid midge^b^^,^^e^	*Clinotanypus claripennis*	Chironomidae	Diptera	5.0
chironomid midge^b^^,^^e^	*Conochironomus acutistus*	Chironomidae	Diptera	5.0
chironomid midge^b^^,^^c^^,^^e^	*Nilodorum brevibucca*	Chironomidae	Diptera	4.5
clubionid spider^b^^,^^d^	*Clubiona*	Culbionidae	Araneae	4.0
cockroach^b^^,^^d^	unidentified	Blatellidae	Blattodea	4.0
cricket^a^^,^^c^	*Acheta domesticus*	Gryllidae	Orthoptera	4.0
fruit fly^a^^,^^c^	*Ceratitis capitata*	Tephritidae	Diptera	4.5
ghost midge^b^^,^^c^^,^^e^	*Chaoborus* sp.	Chaoboridae	Diptera	4.5
green leaf hopper^a^^,^^d^	*Nephotettix nigropictus*	Cicadellidae	Hemiptera	4.0
hersiliid spider^b^^,^^d^	*Hersilia caudata*	Hersiliidae	Araneae	3.0
house fly^a^^,^^d^	*Musca domestica*	Muscidae	Diptera	6.0
jumping spider^b^^,^^d^^,^^e^	*Natta horizontalis*	Salticidae	Araneae	3.0
long-legged fly^b^^,^^d^	unidentified	Dolichopodidae	Diptera	5.0
mantis^b^^,^^d^	unidentified	Mantidae	Mantodea	4.5
mayfly^b^^,^^c^	unidentified	Baetidae	Ephemeroptera	4.5
moth fly^b^^,^^d^	unidentified	Psychodidae	Diptera	3.0
mosquito^a^^,^^c^^,^^d^	*Anopheles gambiae s*.*s*.	Culicidae	Diptera	4.5
mosquito^a^^,^^c^	*Culex quinquefasciatus*	Culicidae	Diptera	4.5
nephilid spider^a^^,^^d^^,^^e^	*Nephilengys*	Nephilididae	Araneae	4.0
oecobiid spider^b^^,^^c^^,^^e^	*Oecobius amboseli*	Oecobidae	Araneae	3.0
vinegar fly^a^^,^^c^	*Drosophila melanogaster*	Drosophilidae	Diptera	3.0
whitefly^b^^,^^d^	unidentified	Aleyrodidae	Hemiptera	2.0
wolf spider^b^^,^^d^^,^^e^	*Pardosa messingerae*	Lycosidae	Araneae	3.0

^a^From stock cultures (see [11,23]).

^b^Collected as needed from Mbita Point field site.

^c^Used in complete series.

^d^Used in mosquito series.

^e^Used in non-mosquito series.

Besides the six mosquito types, we used 29 non-mosquito prey species as lures (table 3), making a total of 35 prey types. Using these lures, we carried out three series of experiments (‘complete’, ‘mosquito’ and ‘non-mosquito’) and, for all experiments, we adopted the simultaneous-presentation testing protocol from Nelson & Jackson [12]. In the complete series, we used nine of the non-mosquito prey species as well as the six mosquito types, with each of the nine non-mosquito species being paired with each other and also with each mosquito type. Data from pairing most of the different mosquito types were already available from an earlier study (table 2) [12] and, after presenting our new findings from the complete series, we added these earlier findings to derive a fuller profile of *E*. *culicivora*'s preferences. For this, the only data from Nelson & Jackson [12] that we used came from simultaneous-presentation testing. However, we provide data here for two important cells (table 2) that were missing from the earlier study (blood *Anopheles* females paired with *Culex* males and blood *Culex* females paired with *Anopheles* males; table 2).

In the mosquito series, we used blood and no-blood female *Anopheles*, with both mosquito types being paired with the 20 non-mosquito prey species that were not used in the complete series and had not been used in any of the previous studies (table 1). In the non-mosquito series, where we used six midge species and four spider species, we paired each midge species with each other midge species and paired each spider species with each other spider species. The six midge species had been paired with mosquitoes in previous experiments (table 1) [11], but not with each other, and they were used as stationary lures in that study but as moving lures here.

Each individual used for making a lure had been preserved in 80% ethanol. To make lures, we positioned the prey item in life-like posture on the centre of a cork disc (thickness 2 mm, diameter slightly more than the body length of the prey). For preservation, the prey item and the cork disc were then sprayed with a transparent plastic adhesive (Crystal Clear Lacquer, Atsco Australia Pty). Further information about making lures can be found elsewhere [1,11]. There is now a vast literature on using lures to investigate salticid prey-choice behaviour [10], with there being no evidence to suggest that these procedures for making lures seriously distort prey appearance in a way that alters preference.

### 2.3. Experimental procedures

**Figure 1.**
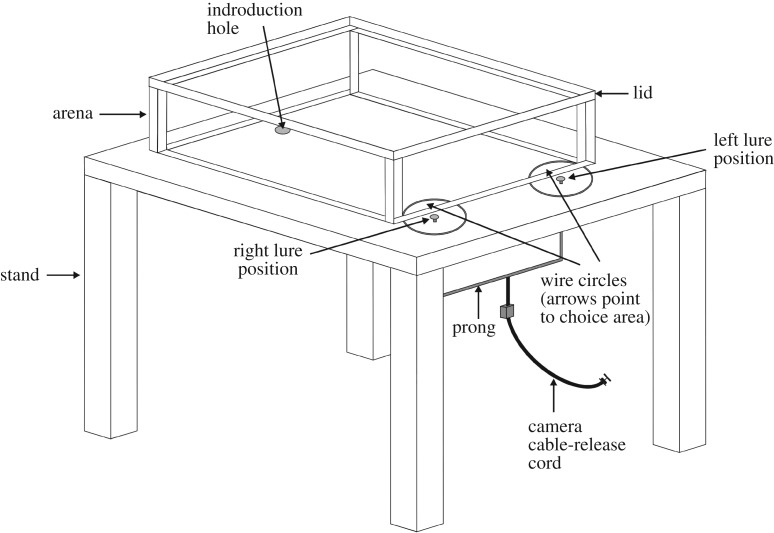
Prey-choice apparatus used for determining the preference profile of *Evarcha culicivora*. Rectangular glass arena with glass lid, sitting on top of Plexiglas stand. Test spider entered arena through the introduction hole. Two lures presented simultaneously, with one being in the left lure position and the other in the right lure position. Movement of lures controlled by using a camera release cord and metal prong. A wire circle surrounded each lure and extended underneath the arena, with the ‘choice area’ being the semicircular region within the arena.

The apparatus was an arena (figure 1) (140 × 115 mm, 35 mm high) made from transparent glass sitting centred on top of a plastic stand (190 × 165, 150 high). There was an ‘introduction hole’ (diameter: 8 mm) in the arena floor and a matching hole in the plastic stand. A rubber bung in the introduction hole could be removed temporarily to let the test spider enter the arena. The introduction hole was situated with its closer side 10 mm from one of the narrow ends of the arena and, outside the opposite narrow end of the arena, there was a ‘left lure hole’ and a ‘right lure hole’ (diameter of each, 5 mm). A lure of one type was centred on top of the right hole and a lure of another type was centred on the top of the left hole, with the side for each lure being decided at random for every trial. The lure stayed in place because the diameter of the hole in the stand was less than the diameter of cork disc that held the prey item. With this arrangement, a test spider inside the arena could see, but not contact or smell, the lures.

A metal prong attached to a camera cable-release cord was connected to the underside of each of the two cork discs. When we pressed the cable-release, the two lures moved in unison 5 mm upward and then, when we released the cable 0.5 s later, the lures moved back to the floor. As soon as the test spider entered the arena, we began a procedure of using the cable-release device for moving the pair of lures up and down once every 30 s.

There were two circles (diameter of 36 mm) made from thin copper wire, with the lure hole on the left being at the centre of one circle and with the lure hole on the right being at the centre of the other circle. Part of each wire circle extended under the arena, with this part being defined as the ‘choice area’ for the particular lure inside that circle. Owing to the glass being transparent, the choice areas were visible to the experimenter when the arena was viewed from above. A trial began after the test spider entered the arena, after which it was allowed 15 min to choose a lure by entering one of the two choice areas.

As in earlier experiments [12], one of our criteria for recording a ‘choice’ was seeing a test spider enter the choice area with its gaze fixated on a lure. We used the term ‘fixation’ for instances in which the corneal lenses of a salticid's large forward-facing principal eyes (see [9]) were held oriented toward a lure (see [8]). There were rare occasions when the 15 min test period ended with the test spider still outside the choice area, but with its gaze fixated on a lure and, on these occasions, we extended the test period until the test spider either made a choice or turned away.

We adopted another two criteria as a step toward being more confident that, when we recorded a choice, it actually was an instance of the test spider actively choosing between the two lures. These criteria had not been used in previous experiments (table 1). One of these was that the test spider had to fixate its gaze at least once on each lure and then maintain continuous fixation on this lure for at least 10 s. The other new criterion was that, while at least 20 mm away from the nearest perimeter of the corresponding choice area, the test spider had to fixate its gaze on the lure that it chose and then maintain continuous fixation on this lure until it had walked inside that choice area.

As another prerequisite for a successful trial, we confirmed that the test spider was hungry. Immediately after making its choice, the test spider was transferred to a cylindrical plastic rearing cage (diameter 55 mm, height 100 mm); 30 min later, three midges (*N*. *brevibucca*) were put in the cage with the spider. Whenever a test spider failed to capture one of the midges during a 60 min interval, we ignored the data from the trial in which this test spider had been used and we did not use this test spider again. The rationale for this criterion was to limit our data to instances in which it would be especially reasonable to conclude that the test spider had been motivated to capture prey when it chose a lure.

Nelson & Jackson [15] had adopted a similar, but stricter, criterion for accepting trials as successful. The day after an experimental trial using two lures or two virtual prey, a live-prey trial was initiated using the same two prey types. The data for an experimental trial were only accepted if the test spider had made the same choice when tested with living prey as it had with lures or virtual prey. The variety of prey we used in this study meant that adopting this stricter criterion would have been prohibitively difficult. However, choices in that study were usually consistent, which suggests our findings would not have been especially different if we had used this stricter criterion. It is also relevant that consistency of choice between lure trials and live-prey trials in the earlier study [15] can be envisaged as confirmation that our testing methods based on using lures alone (only visual stimuli) reveal the same preferences *E*. *culicivora* expresses when having access to living prey accompanied by the stimuli from other sensorymodalities.

All testing was carried out between 08.00 and 14.00 (laboratory photoperiod 12 L : 12 D, lights on at 07.00) and no test spider or lure was used more than once. In addition to ambient lighting from fluorescent ceiling lamps, the apparatus was lit from 400 mm overhead by a 100 W incandescent lamp. Between trials, the apparatus was washed with 80% ethanol followed by distilled water and then dried.

In this study, where our objective was to detect variation in preference strength across a much wider range of prey types than had been considered in the past, it was not realistic to adopt all of the experimental protocols and pre-trial fasting durations used by Nelson & Jackson [12]. As a compromise, we used only simultaneous-presentation testing, this being the testing protocol that, in the earlier study, appeared to be the most effective for detecting preferences and, as each trial was on a single day instead of on two successive days, it was the simplest method to use. As another compromise, only two fasting durations (7-day and 1-day) were used, these being the fasting durations that were the most effective for discriminating between preference strengths in the previous study [12].

In the complete series, each test spider had fasted for 7 days before being used in a trial in which two prey types were present and then, whenever findings were not significant after the 7-day fast, we carried out another experiment using the same pair of prey types, but this time with another set of test spiders that had fasted for only 1 day. We used only 7-day fasting for the mosquito series and the mosquitoes in this series were always *Anopheles* females. The rationale for this decision was that, based on findings from the complete series and from earlier research (table 1), we expected to find a strong preference for female *Anopheles* mosquitoes. We used only 1-day fasting in the non-mosquito series because, for this series, our objective was only to detect whether a preference was present (i.e. as our objective here did not include measuring the strengths of preferences, we used the fasting duration that was most effective at detecting even a weak preference). For each experiment, we continued testing until we had a specified number of successful trials (30 for each experiment in the complete series; 25 for each experiment in the mosquito and non-mosquito series). Data were then analysed using *χ*^2^ tests of goodness of fit (null hypothesis: equal likelihood of choosing each prey type).

We use the expression ‘strong preference’ when, for a given prey pair in the complete series, significantly more test spiders chose one instead of the other after a 7-day fast. We use the expression ‘weak preference’ when, for a given pair in the complete series, significantly more test spiders chose one instead of the other after a 1-day fast, but not after the 7-day fast. We use the expression ‘no preference’ for instances in which there was no significant tendency to choose one prey type instead of the other after the 7-day or after the 1-day fast.

Wanting a sharp distinction between weak and strong preference, we decided to set *α* at 0.01, with this decision also being influenced by there being especially many prey-choice experiments in this study. However, instances of an experimental outcome being not significant with *α* set at 0.01 when it would have been significant if *α* had instead been 0.05 were rare and, when it did happen, the consequences of *α* being 0.01 instead of 0.05 were only that preference strength was recorded as weak instead of strong (i.e. it never entailed a change from preference present to nil preference).

## 3. Results

### 3.1. Prey records from the field

We accumulated 1115 records of *E*. *culicivora* feeding on prey in the field (tables 4 and 5) and there was no striking variation related to whether the *E*. *culicivora* individuals were adult females, adult males or juveniles. Mosquitoes accounted for 80.2% of the 1115 records, with midges being the second most common prey type (9.2%). After midges, the next most common prey type (5.0% of the 1115 records) was ‘other dipterans’ (i.e. non-mosquito and non-midge species from the order Diptera).

**Table 4.** Records of prey on which adult females, adult males and juveniles of *Evarcha culicivora* were found feeding in the field. Each prey type listed, when possible, by common name, genus, species, order and family. Sex of mosquitoes and whether female mosquitoes were carrying blood indicated. Blood female: there was evidence that these mosquitoes were carrying blood. No-blood female: there was no evidence that these mosquitoes were carrying blood.

order	description	genus, species	family	records for adult females^a^	records for adult males^b^	records for juveniles^c^	all records^d^
Diptera	blood female anopheline mosquito	*Anopheles*	Culicidae	12 (2.7%)	9 (2.6%)	8 (2.4%)	29 (2.6%)
Diptera	blood female culicine mosquito	*Culex*	Culicidae	19 (4.3%)	13 (3.8%)	5 (1.5%)	37 (3.3%)
Diptera	blood female culicine mosquito	*Aedes*	Culicidae	6 (1.3%)	5 (1.5%)	4 (1.2%)	15 (1.3%)
Diptera	unidentified blood female mosquito	unidentified	Culicidae	4 (0.9%)	7 (2.0%)	8 (2.4%)	19 (1.7%)
Diptera	no-blood female anopheline mosquito	*Anopheles*	Culicidae	91 (20.4%)	61 (17.8%)	76 (23.2%)	228 (20.4%)
Diptera	no-blood female culicine mosquito	*Culex*	Culicidae	49 (11.0%)	31 (9.1%)	28 (8.5%)	108 (9.7%)
Diptera	no-blood female culicine mosquito	*Aedes*	Culicidae	46 (10.3%)	17 (5.0%)	29 (8.8%)	92 (8.3%)
Diptera	unidentified no-blood female mosquito	unidentified	Culicidae	78 (17.5%)	68 (19.9%)	68 (20.7%)	214 (19.2%)
Diptera	male anopheline mosquito	*Anopheles*	Culicidae	22 (4.9%)	9 (2.6%)	8 (2.4%)	39 (3.5%)
Diptera	male culicine mosquito	*Culex*	Culicidae	11 (2.5%)	4 (1.2%)	12 (3.7%)	27 (2.4%)
Diptera	male culicine mosquito	*Aedes*	Culicidae	10 (2.2%)	17 (5.0%)	10 (3.0%)	37 (3.3%)
Diptera	unidentified male mosquito	unidentified	Culicidae	23 (5.2%)	14 (4.1%)	13 (4.0%)	50 (4.5%)
Diptera	ghost midge	*Chaoborus*	Chaoboridae	13 (2.9%)	8 (2.3%)	3 (0.9%)	24 (2.2%)
Diptera	chironomid midge	unidentified	Chironomidae	22 (4.9%)	33 (9.6%)	24 (7.3%)	79 (7.1%)
Diptera	moth fly	unidentified	Psychodidae	3 (0.7%)	4 (1.2%)	5 (1.5%)	12 (1.1%)
Diptera	long-legged fly	unidentified	Dolichopodidae	2 (0.4%)	1 (0.3%)	1 (0.3%)	4 (0.4%)
Diptera	unidentified fly	unidentified	unidentified	8 (1.8%)	22 (6.4%)	10 (3.0%)	40 (3.6%)
Araneae	conspecific juvenile	*Evarcha culicivora*	Salticidae	2 (0.4%)	3 (0.9%)	0	5 (0.4%)
Araneae	opposite-sex conspecific adult	*Evarcha culicivora*	Salticidae	1 (0.2%)	1 (0.3%)	0	2 (0.2%)
Araneae	jumping spider	*Natta*	Salticidae	1 (0.2%)	0	1 (0.3%)	2 (0.2%)
Araneae	unidentified jumping spider	unidentified	Salticidae	2 (0.4%)	1 (0.3%)	0	3 (0.3%)
Araneae	oecobiid spider	*Oecobius amboseli*	Oecobiidae	0	1 (0.3%)	3 (0.9%)	4 (0.4%)
Araneae	wolf spider	unidentified	Lycosidae	1 (0.2%)	2 (0.6%)	1 (0.3%)	4 (0.4%)
Araneae	unidentified non-salticid spider	unidentified	unidentified	1 (0.2%)	1 (0.3%)	1 (0.3%)	3 (0.3%)
Ephemeroptera	mayfly	unidentified	Baetidae	5 (1.1%)	4 (1.2%)	2 (0.6%)	11 (1.0%)
Hemiptera	leafhopper	unidentified	Cicadellidae	2 (0.4%)	1 (0.3%)	1 (0.3%)	4 (0.4%)
Hemiptera	big-eyed bug	*Geocoris*	Geocoridae	1 (0.2%)	0	1 (0.3%)	2 (0.2%)
Hemiptera	mirid bug	unidentified	Miridae	1 (0.2%)	0	1 (0.3%)	2 (0.2%)
Hemiptera	aphid	unidentified	Aphidae	2 (0.4%)	1 (0.3%)	0	3 (0.3%)
Lepidoptera	caterpillar	unidentified	unidentified	1 (0.2%)	0	4 (1.2%)	5 (0.4%)
Mantodea	mantis	unidentified	Mantidae	1 (0.2%)	2 (0.6%)	0	3 (0.3%)
Blattodea	cockroach	unidentified	Blatellidae	1 (0.2%)	0	1 (0.3%)	2 (0.2%)
Hymenoptera	winged ant	unidentified	Formicidae	1 (0.2%)	1 (0.3%)	0	2 (0.2%)
Orthoptera	cricket	unidentified	Gryllidae	2 (0.4%)	0	0	2 (0.2%)
Psocoptera	barklouse	unidentified	unidentified	1 (0.2%)	1 (0.3%)	0	2 (0.2%)

^a^Total 445.

^b^Total 342.

^c^Total 328.

^d^Total 1115.

**Table 5.** Analysis of field records (table 4) of prey on which *Evarcha culicivora* was found feeding.

prey	records for adult females	records for adult males	records for juveniles	total for all *E*.*culicivora*
mosquitoes	371 (83.4%)	255 (74.6%)	269 (82.0%)	895 (80.3%)
female mosquitoes	305 (68.5%)	211 (61.7%)	226 (68.9%)	742 (66.5%)
blood female mosquitoes	41 (9.2%)	34 (9.9%)	25 (7.6%)	100 (9.0%)
no-blood female mosquitoes	264 (59.3%)	177 (51.8%)	201 (61.3%)	642 (57.6%)
male mosquitoes	66 (14.8%)	44 (12.9%)	43 (13.1%)	153 (13.7%)
*Anopheles*	125 (28.1%)	79 (23.1%)	92 (28.0%)	296 (26.5%)
*Culex*	79 (17.8%)	48 (14.0%)	45 (13.7%)	172 (15.4%)
*Aedes*	62 (13.9%)	39 (11.4%)	43 (13.1%)	144 (12.9%)
culicine mosquitoes	141 (31.7%)	87 (25.4%)	88 (26.8%)	316 (28.3%)
unidentified mosquitoes	105 (23.6%)	89 (26.0%)	89 (27.1%)	283 (25.4%)
*Anopheles* females	103 (23.1%)	70 (20.5%)	84 (25.6%)	257 (23.0%)
*Culex* females	68 (15.3%)	44 (12.9%)	33 (10.1%)	145 (13.0%)
*Aedes* females	52 (11.7%)	22 (6.4%)	33 (10.1%)	107 (9.6%)
culicine females	120 (27.0%)	66 (19.3%)	66 (20.1%)	252 (22.6%)
unidentified female mosquitoes	82 (18.4%)	75 (21.9%)	76 (23.2%)	233 (20.9%)
midges	35 (7.9%)	41 (12.0%)	27 (8.2%)	103 (9.2%)
non-mosquito dipterans	48 (10.8%)	68 (19.9%)	43 (13.1%)	159 (14.3%)
Diptera	419 (94.2%)	323 (94.4%)	312 (95.1%)	1054 (94.5%)
non-mosquito and non-midge Diptera	13 (2.9%)	27 (7.9%)	16 (4.9%)	56 (5.0%)
non-mosquito and non-midge Diptera + mayflies	18 (4.0%)	31 (9.1/%)	18 (5.5%)	67 (6.0%)
insects	437 (98.2%)	333 (97.4%)	322 (98.2%)	1092 (97.9%)
non-dipteran insects	18 (4.0%)	10 (2.9%)	10 (3.0%)	38 (3.4%)
non-dipteran insects and non-mayfly insects	13 (2.9%)	6 (1.8%)	8 (2.4%)	27 (2.4%)
spiders	8 (1.8%)	9 (2.6%)	6 (1.8%)	23 (2.1%)
non-mosquito and non-midge prey	39 (8.8%)	46 (13.5%)	32 (9.8%)	117 (10.5%)
total number of records	445 (39.9%)	342 (30.7%)	328 (29.4%)	1115

Prey belonged to 10 arthropod orders (tables 4 and 5). Ranked by prevalence in the records, 94.5% were Diptera, 2.1% Araneae, 1.0% Ephemeroptera, 1.0% Hemiptera, 0.4% Lepidoptera, 0.3% Mantodea, 0.2% Blattodea, 0.2% Hymenoptera, 0.2% Orthoptera and 0.2% Psocoptera. The majority of the 103 midges were chironomids (76.7%). About half (52.2%) of the 23 spiders in the prey records were salticids and seven of the 12 salticids were conspecific individuals (five juveniles being eaten by adults and two adults being eaten by opposite-sex conspecific adults).

The majority of the 895 mosquitoes in the field records (table 5) were adult females (82.9%). Of the 895 mosquitoes, 33.1% were anophelines (genus *Anopheles*), 35.3% were culicines and 31.6% could not be identified to subfamily. Of the 316 culicines, 54.4% were *Culex* and 45.6% were *Aedes*. There was a remarkable consistency in the percentages of mosquitoes that were females: 86.8% of 296 *Anopheles*, 84.3% of 172 *Culex*, 74.3% of 144 *Aedes* and 82.3% of the 283 mosquitoes that could not be identified to subfamily. We confirmed that 13.5% of the 742 female mosquitoes were carrying blood. The percentage of females for which blood was detected was: 11.3% of 257 *Anopheles* females, 25.5% of 145 *Culex* females, 14.0% of 107 *Aedes* females and 8.2% of 233 female mosquitoes that could not be identified to subfamily.

All dipterans were adults and all lepidopterans were larvae (caterpillars). The mayflies and barklice were adults. All of the aphids were probably adults, but the other hemipterans were juveniles. All mantises, cockroaches and crickets were juveniles (nymphs). Oecobiid spiders were a mixture of adults and juveniles, two conspecific individuals were adults, and all other spiders were juveniles.

### 3.2. Choice between mosquito and non-mosquito prey

Adult female and juvenile test spiders from the complete series expressed a consistent strong preference for blood female mosquitoes (*Anopheles* and *Culex*) regardless of which of the nine non-mosquito prey species was used (tables 6 and 7). Juvenile test spiders also expressed a consistent strong preference for no-blood *Anopheles* females regardless of which of the nine alternative prey species was used. Adults differed from juveniles by expressing only a weak preference for no-blood *Anopheles* females when the alternative was a midge, but resembled juveniles by expressing a strong preference when the alternative was any other non-mosquito species.

**Table 6.** Findings for adult females and juveniles of *Evarcha culicivora* (‘test spiders’) in the complete series of prey-choice experiments (see the text). For each experiment (row), simultaneous-presentation testing was used. *n* = 30 test spiders for each row. See table 3 for details pertaining to prey and text for methods. Chironomid midge: *Nilodorum brevibucca*. Experiments with 1-day pre-trial fasts carried out only when findings were not significant after 7-day fast. Data analysis: test of goodness of fit (null hypothesis: as likely to choose prey 2 as to choose prey 1).

				adult female test spiders		juvenile test spiders	
prey 1	prey 2	diet	fast	chose prey 1	test of goodness of fit	chose prey 1	test of goodness of fit
blood *Anopheles* female	*Culex* male	standard	7-day	30	*χ*^2^ = 30.00, *p *< 0.001	28	*χ*^2^ = 22.53, *p *< 0.001
blood *Anopheles* female	ghost midge	standard	7-day	28	*χ*^2^ = 22.53, *p *< 0.001	29	*χ*^2^ = 26.13, *p *< 0.001
blood *Anopheles* female	ghost midge	spider-only	7-day	—	—	30	*χ*^2^ = 30.00, *p *< 0.001
blood *Anopheles* female	chironomid midge	standard	7-day	24	*χ*^2^ = 10.80, *p* = 0.001	27	*χ*^2^ = 19.20, *p *< 0.001
blood *Anopheles* female	vinegar fly	standard	7-day	29	*χ*^2^ = 26.13, *p *< 0.001	24	*χ*^2^ = 10.80, *p* = 0.001
blood *Anopheles* female	mayfly	standard	7-day	25	*χ*^2^ = 13.33, *p *< 0.001	27	*χ*^2^ = 19.20, *p *< 0.001
blood *Anopheles* female	fruit fly	standard	7-day	28	*χ*^2^ = 22.53, *p *< 0.001	29	*χ*^2^ = 26.13, *p *< 0.001
blood *Anopheles* female	cricket	standard	7-day	27	*χ*^2^ = 19.20, *p *< 0.001	30	*χ*^2^ = 30.00, *p *< 0.001
blood *Anopheles* female	caterpillar	standard	7-day	25	*χ*^2^ = 13.33, *p *< 0.001	28	*χ*^2^ = 22.53, *p *< 0.001
blood *Anopheles* female	aphid	standard	7-day	27	*χ*^2^ = 19.20, *p *< 0.001	27	*χ*^2^ = 19.20, *p *< 0.001
blood *Anopheles* female	oecobiid spider	standard	7-day	29	*χ*^2^ = 26.13, *p *< 0.001	28	*χ*^2^ = 22.53, *p *< 0.001
blood *Anopheles* female	oecobiid spider	spider-only	7-day	—	—	30	*χ*^2^ = 30.00, *p *< 0.001
blood *Culex* female	*Anopheles* male	standard	7-day	25	*χ*^2^ = 13.33, *p *< 0.001	12	*χ*^2^ = 1.20, *p* = 0.273
blood *Culex* female	*Anopheles* male	standard	1-day	—	—	7	*χ*^2^ = 8.53^a^, *p* = 0.003
blood *Culex* female	ghost midge	standard	7-day	29	*χ*^2^ = 26.13, *p *< 0.001	30	*χ*^2^ = 30.00, *p *< 0.001
blood *Culex* female	chironomid midge	standard	7-day	26	*χ*^2^ = 16.13, *p *< 0.001	30	*χ*^2^ = 30.00, *p *< 0.001
blood *Culex* female	vinegar fly	standard	7-day	27	*χ*^2^ = 19.20, *p *< 0.001	28	*χ*^2^ = 22.53, *p *< 0.001
blood *Culex* female	mayfly	standard	7-day	30	*χ*^2^ = 30.00, *p *< 0.001	29	*χ*^2^ = 26.13, *p *< 0.001
blood *Culex* female	fruit fly	standard	7-day	27	*χ*^2^ = 19.20, *p *< 0.001	30	*χ*^2^ = 30.00, *p *< 0.001
blood *Culex* female	cricket	standard	7-day	30	*χ*^2^ = 30.00, *p *< 0.001	26	*χ*^2^ = 16.13, *p *< 0.001
blood *Culex* female	caterpillar	standard	7-day	25	*χ*^2^ = 13.33, *p *< 0.001	26	*χ*^2^ = 16.13, *p *< 0.001
blood *Culex* female	aphid	standard	7-day	30	*χ*^2^ = 30.00, *p *< 0.001	30	*χ*^2^ = 30.00, *p *< 0.001
blood *Culex* female	oecobiid spider	standard	7-day	28	*χ*^2^ = 22.53, *p *< 0.001	25	*χ*^2^ = 13.33, *p *< 0.001
no-blood *Anopheles* female	ghost midge	standard	7-day	21	*χ*^2^ = 4.80, *p* = 0.028	25	*χ*^2^ = 13.33, *p *< 0.001
no-blood *Anopheles* female	ghost midge	standard	1-day	27	*χ*^2^ = 19.20, *p *< 0.001	—	—
no-blood *Anopheles* female	ghost midge	spider-only	7-day	—	—	26	*χ*^2^ = 16.13, *p *< 0.001
no-blood *Anopheles* female	chironomid midge	standard	7-day	17	*χ*^2^ = 0.53, *p* = 0.465	24	*χ*^2^ = 10.80, *p* = 0.001
no-blood *Anopheles* female	chironomid midge	standard	1-day	26	*χ*^2^ = 16.13, *p *< 0.001	—	—
no-blood *Anopheles* female	vinegar fly	standard	7-day	25	*χ*^2^ = 13.33, *p *< 0.001	28	*χ*^2^ = 22.53, *p *< 0.001
no-blood *Anopheles* female	mayfly	standard	7-day	26	*χ*^2^ = 16.13, *p *< 0.001	26	*χ*^2^ = 16.13, *p *< 0.001
no-blood *Anopheles* female	fruit fly	standard	7-day	26	*χ*^2^ = 16.13, *p *< 0.001	29	*χ*^2^ = 26.13, *p *< 0.001
no-blood *Anopheles* female	cricket	standard	7-day	30	*χ*^2^ = 30.00, *p *< 0.001	27	*χ*^2^ = 19.20, *p *< 0.001
no-blood *Anopheles* female	caterpillar	standard	7-day	30	*χ*^2^ = 30.00, *p *< 0.001	26	*χ*^2^ = 16.13, *p *< 0.001
no-blood *Anopheles* female	aphid	standard	7-day	27	*χ*^2^ = 19.20, *p *< 0.001	28	*χ*^2^ = 22.53, *p *< 0.001
no-blood *Anopheles* female	oecobiid spider	standard	7-day	25	*χ*^2^ = 13.33, *p *< 0.001	29	*χ*^2^ = 26.13, *p *< 0.001
no-blood *Anopheles* female	oecobiid spider	spider-only	7-day	—	—	25	*χ*^2^ = 13.33, *p *< 0.001
no-blood *Culex* female	ghost midge	standard	7-day	15	*χ*^2^ = 0.00, *p* = 1	16	*χ*^2^ = 0.13, *p* = 0.715
no-blood *Culex* female	ghost midge	standard	1-day	17	*χ*^2^ = 0.53, *p* = 0.465	14	*χ*^2^ = 0.13, *p* = 0.715
no-blood *Culex* female	chironomid midge	standard	7-day	14	*χ*^2^ = 0.13, *p* = 0.715	15	*χ*^2^ = 0.00, *p* = 1
no-blood *Culex* female	chironomid midge	standard	1-day	12	*χ*^2^ = 1.20, *p* = 0.273	15	*χ*^2^ = 0.00, *p* = 1
no-blood *Culex* female	vinegar fly	standard	7-day	15	*χ*^2^ =0.00, *p* = 1	16	*χ*^2^ = 0.13, *p* = 0.715
no-blood *Culex* female	vinegar fly	standard	1-day	26	*χ*^2^ = 16.13, *p *< 0.001	29	*χ*^2^ = 26.13, *p *< 0.001
no-blood *Culex* female	mayfly	standard	7-day	22	*χ*^2^ = 6.53, *p* = 0.011	12	*χ*^2^ = 1.20, *p* = 0.273
no-blood *Culex* female	mayfly	standard	1-day	25	*χ*^2^ = 13.33, *p *< 0.001	28	*χ*^2^ = 22.53, *p *< 0.001
no-blood *Culex* female	fruit fly	standard	7-day	30	*χ*^2^ = 30.00, *p *< 0.001	29	*χ*^2^ = 26.13, *p *< 0.001
no-blood *Culex* female	cricket	standard	7-day	25	*χ*^2^ = 13.33, *p *< 0.001	24	*χ*^2^ = 10.80, *p* = 0.001
no-blood *Culex* female	caterpillar	standard	7-day	30	*χ*^2^ = 30.00, *p *< 0.001	25	*χ*^2^ = 13.33, *p *< 0.001
no-blood *Culex* female	aphid	standard	7-day	24	*χ*^2^ = 10.80, *p* = 0.001	28	*χ*^2^ = 22.53, *p *< 0.001
no-blood *Culex* female	oecobiid spider	standard	7-day	30	*χ*^2^ = 30.00, *p *< 0.001	30	*χ*^2^ = 30.00, *p *< 0.001
*Anopheles* male	ghost midge	standard	7-day	19	*χ*^2^ = 2.13, *p* = 0.144	15	*χ*^2^ = 0.00, *p* = 1
*Anopheles* male	ghost midge	standard	1-day	10	*χ*^2^ = 3.33, *p* = 0.068	27	*χ*^2^ = 19.20, *p *< 0.001
*Anopheles* male	ghost midge	spider-only	7-day	—	—	13	*χ*^2^ = 0.53, *p* = 0.465
*Anopheles* male	ghost midge	spider-only	1-day	—	—	28	*χ*^2^ = 22.53, *p *< 0.001
*Anopheles* male	chironomid midge	standard	7-day	13	*χ*^2^ = 0.53, *p* = 0.465	21	*χ*^2^ = 4.80, *p* = 0.028
*Anopheles* male	chironomid midge	standard	1-day	11	*χ*^2^ = 2.13, *p* = 0.144	29	*χ*^2^ = 26.13, *p *< 0.001
*Anopheles* male	vinegar fly	standard	7-day	14	*χ*^2^ = 0.13, *p* = 0.715	16	*χ*^2^ = 0.13, *p* = 0.715
*Anopheles* male	vinegar fly	standard	1-day	24	*χ*^2^ = 10.80, *p* = 0.001	26	*χ*^2^ = 16.13, *p *< 0.001
*Anopheles* male	mayfly	standard	7-day	17	*χ*^2^ = 0.53, *p* = 0.465	15	*χ*^2^ = 0.00, *p* = 1
*Anopheles* male	mayfly	standard	1-day	28	*χ*^2^ = 22.53, *p *< 0.001	28	*χ*^2^ = 22.53, *p *< 0.001
*Anopheles* male	fruit fly	standard	7-day	23	*χ*^2^ = 8.53, *p* = 0.003	27	*χ*^2^ = 19.20, *p *< 0.001
*Anopheles* male	cricket	standard	7-day	26	*χ*^2^ = 16.13, *p *< 0.001	26	*χ*^2^ = 16.13, *p *< 0.001
*Anopheles* male	caterpillar	standard	7-day	25	*χ*^2^ = 13.33, *p *< 0.001	28	*χ*^2^ = 22.53, *p *< 0.001
*Anopheles* male	aphid	standard	7-day	30	*χ*^2^ = 30.00, *p *< 0.001	25	*χ*^2^ = 13.33, *p *< 0.001
*Anopheles* male	oecobiid spider	standard	7-day	25	*χ*^2^ = 13.33, *p *< 0.001	29	*χ*^2^ = 26.13, *p *< 0.001
*Anopheles* male	oecobiid spider	spider-only	7-day	—	—	27	*χ*^2^ = 19.20, *p *< 0.001
*Culex* male	ghost midge	standard	7-day	20	*χ*^2^ = 3.33, *p* = 0.068	15	*χ*^2^ = 0.00, *p* = 1
*Culex* male	ghost midge	standard	1-day	12	*χ*^2^ = 1.20, *p* = 0.273	15	*χ*^2^ = 0.00, *p* = 1
*Culex* male	chironomid midge	standard	7-day	15	*χ*^2^ = 0.00, *p* = 1	11	*χ*^2^ = 2.13, *p* = 0.144
*Culex* male	chironomid midge	standard	1-day	16	*χ*^2^ = 0.13, *p* = 0.715	18	*χ*^2^ = 1.20, *p* = 0.273
*Culex* male	vinegar fly	standard	7-day	15	*χ*^2^ = 0.00, *p* = 1	22	*χ*^2^ = 6.53, *p* = 0.011
*Culex* male	vinegar fly	standard	1-day	24	*χ*^2^ = 10.80, *p* = 0.001	15	*χ*^2^ = 0.00, *p* = 1
*Culex* male	mayfly	standard	7-day	14	*χ*^2^ = 0.13, *p* = 0.715	21	*χ*^2^ = 4.80, *p* = 0.028
*Culex* male	mayfly	standard	1-day	28	*χ*^2^ = 22.53, *p *< 0.001	24	*χ*^2^ = 10.80, *p* = 0.001
*Culex* male	fruit fly	standard	7-day	24	*χ*^2^ = 10.80, *p* = 0.001	29	*χ*^2^ = 26.13, *p *< 0.001
*Culex* male	cricket	standard	7 day	27	*χ*^2^ = 19.20, *p *< 0.001	24	*χ*^2^ = 10.80, *p* = 0.001
*Culex* male	caterpillar	standard	7-day	29	*χ*^2^ = 26.13, *p *< 0.001	28	*χ*^2^ = 22.53, *p *< 0.001
*Culex* male	aphid	standard	7-day	24	*χ*^2^ = 10.80, *p* = 0.001	25	*χ*^2^ = 13.33, *p *< 0.001
*Culex* male	oecobiid spider	standard	7-day	30	*χ*^2^ = 30.00, *p *< 0.001	24	*χ*^2^ = 10.80, *p* = 0.001
ghost midge	chironomid midge	standard	7-day	16	*χ*^2^ = 0.13, *p* = 0.715	16	*χ*^2^ = 0.13, *p* = 0.715
ghost midge	chironomid midge	standard	1 day	12	*χ*^2^ = 1.20, *p* = 0.273	26	*χ*^2^ = 16.13, *p *< 0.001
ghost midge	vinegar fly	standard	7-day	12	*χ*^2^ = 1.20, *p* = 0.273	16	*χ*^2^ = 0.13, *p* = 0.715
ghost midge	vinegar fly	standard	1-day	24	*χ*^2^ = 10.80, *p* = 0.001	27	*χ*^2^ = 19.20, *p *< 0.001
ghost midge	mayfly	standard	7-day	14	*χ*^2^ = 0.13, *p* = 0.715	12	*χ*^2^ = 1.20, *p* = 0.273
ghost midge	mayfly	standard	1-day	29	*χ*^2^ = 26.13, *p *< 0.001	29	*χ*^2^ = 26.13, *p *< 0.001
ghost midge	fruit fly	standard	7-day	23	*χ*^2^ = 8.53, *p* = 0.003	27	*χ*^2^ = 19.20, *p *< 0.001
ghost midge	cricket	standard	7 day	28	*χ*^2^ = 22.53, *p *< 0.001	26	*χ*^2^ = 16.13, *p *< 0.001
ghost midge	caterpillar	standard	7 day	27	*χ*^2^ = 19.20, *p *< 0.001	27	*χ*^2^ = 19.20, *p *< 0.001
ghost midge	aphid	standard	7 day	30	*χ*^2^ = 30.00, *p *< 0.001	29	*χ*^2^ = 26.13, *p *< 0.001
ghost midge	oecobiid spider	standard	7-day	28	*χ*^2^ = 22.53, *p *< 0.001	26	*χ*^2^ = 16.13, *p *< 0.001
ghost midge	oecobiid spider	spider-only	7-day	—	—	27	*χ*^2^ = 19.20, *p *< 0.001
chironomid midge	vinegar fly	standard	7-day	13	*χ*^2^ = 0.53, *p* = 0.465	13	*χ*^2^ = 0.53, *p* = 0.465
chironomid midge	vinegar fly	standard	1-day	23	*χ*^2^ = 8.53, *p* = 0.003	27	*χ*^2^ = 19.20, *p *< 0.001
chironomid midge	mayfly	standard	7-day	15	*χ*^2^ = 0.00, *p* = 1	12	*χ*^2^ = 1.20, *p* = 0.273
chironomid midge	mayfly	standard	1-day	25	*χ*^2^ = 13.33, *p *< 0.001	26	*χ*^2^ = 16.13, *p *< 0.001
chironomid midge	fruit fly	standard	7-day	27	*χ*^2^ = 19.20, *p *< 0.001	27	*χ*^2^ = 19.20, *p *< 0.001
chironomid midge	cricket	standard	7-day	26	*χ*^2^ = 16.13, *p *< 0.001	29	*χ*^2^ = 26.13, *p *< 0.001
chironomid midge	caterpillar	standard	7-day	28	*χ*^2^ = 22.53, *p *< 0.001	30	*χ*^2^ = 30.00, *p *< 0.001
chironomid midge	aphid	standard	7-day	29	*χ*^2^ = 26.13, *p *< 0.001	26	*χ*^2^ = 16.13, *p *< 0.001
chironomid midge	oecobiid spider	standard	7-day	27	*χ*^2^ = 19.20, *p *< 0.001	28	*χ*^2^ = 22.53, *p *< 0.001
vinegar fly	mayfly	standard	7-day	12	*χ*^2^ = 1.20, *p* = 0.273	16	*χ*^2^ = 0.13, *p* = 0.715
vinegar fly	mayfly	standard	1-day	13	*χ*^2^ = 0.53, *p* = 0.465	17	*χ*^2^ = 0.53, *p* = 0.465
vinegar fly	fruit fly	standard	7-day	18	*χ*^2^ = 1.20, *p* = 0.273	16	*χ*^2^ = 0.13, *p* = 0.715
vinegar fly	fruit fly	standard	1-day	15	*χ*^2^ = 0.00, *p* = 1	13	*χ*^2^ = 0.53, *p* = 0.465
vinegar fly	cricket	standard	7-day	29	*χ*^2^ = 26.13, *p *< 0.001	27	*χ*^2^ = 19.20, *p *< 0.001
vinegar fly	caterpillar	standard	7-day	26	*χ*^2^ = 16.13, *p *< 0.001	26	*χ*^2^ = 16.13, *p *< 0.001
vinegar fly	aphid	standard	7-day	25	*χ*^2^ = 13.33, *p *< 0.001	26	*χ*^2^ = 16.13, *p *< 0.001
vinegar fly	oecobiid spider	standard	7-day	23	*χ*^2^ = 8.53, *p* = 0.003	29	*χ*^2^ = 26.13, *p *< 0.001
vinegar fly	oecobiid spider	spider-only	7-day	—	—	24	*χ*^2^ = 10.80, *p* = 0.001
mayfly	fruit fly	standard	7-day	15	*χ*^2^ = 0.00, *p* = 1	13	*χ*^2^ = 0.53, *p* = 0.465
mayfly	fruit fly	standard	1-day	10	*χ*^2^ = 3.33, *p* = 0.068	18	*χ*^2^ = 1.20, *p* = 0.273
mayfly	cricket	standard	7-day	15	*χ*^2^ = 0.00, *p* = 1	20	*χ*^2^ = 3.33, *p* = 0.068
mayfly	cricket	standard	1-day	29	*χ*^2^ = 26.13, *p *< 0.001	30	*χ*^2^ = 30.00, *p *< 0.001
mayfly	caterpillar	standard	7-day	30	*χ*^2^ = 30.00, *p *< 0.001	28	*χ*^2^ = 22.53, *p *< 0.001
mayfly	aphid	standard	7-day	27	*χ*^2^ = 19.20, *p *< 0.001	27	*χ*^2^ = 19.20, *p *< 0.001
mayfly	oecobiid spider	standard	7-day	28	*χ*^2^ = 22.53, *p *< 0.001	25	*χ*^2^ = 13.33, *p *< 0.001
fruit fly	cricket	standard	7-day	25	*χ*^2^ = 13.33, *p *< 0.001	17	*χ*^2^ = 0.53, *p* = 0.465
fruit fly	cricket	standard	1-day	—	—	25	*χ*^2^ = 13.33, *p *< 0.001
fruit fly	caterpillar	standard	7-day	27	*χ*^2^ = 19.20, *p *< 0.001	28	*χ*^2^ = 22.53, *p *< 0.001
fruit fly	aphid	standard	7-day	26	*χ*^2^ = 16.13, *p *< 0.001	29	*χ*^2^ = 26.13, *p *< 0.001
fruit fly	oecobiid spider	standard	7-day	29	*χ*^2^ = 26.13, *p *< 0.001	26	*χ*^2^ = 16.13, *p *< 0.001
cricket	caterpillar	standard	7-day	21	*χ*^2^ = 4.80, *p* = 0.028	15	*χ*^2^ = 0.00, *p* = 1
cricket	caterpillar	standard	1-day	14	*χ*^2^ = 0.13, *p* = 0.715	17	*χ*^2^ = 0.53, *p* = 0.465
cricket	aphid	standard	7-day	14	*χ*^2^ = 0.13, *p* = 0.715	16	*χ*^2^ = 0.13, *p* = 0.715
cricket	aphid	standard	1-day	15	*χ*^2^ = 0.00, *p* = 1	12	*χ*^2^ = 1.20, *p* = 0.273
cricket	oecobiid spider	standard	7-day	13	*χ*^2^ = 0.53, *p* = 0.465	10	*χ*^2^ = 3.33, *p* = 0.068
cricket	oecobiid spider	standard	1-day	15	*χ*^2^ = 0.00, *p* = 1	16	*χ*^2^ = 0.13, *p* = 0.715
caterpillar	aphid	standard	7-day	16	*χ*^2^ = 0.13, *p* = 0.715	14	*χ*^2^ = 0.13, *p* = 0.715
caterpillar	aphid	standard	1-day	15	*χ*^2^ = 0.00, *p* = 1	14	*χ*^2^ = 0.13, *p* = 0.715
caterpillar	oecobiid spider	standard	7-day	11	*χ*^2^ = 2.13, *p* = 0.144	17	*χ*^2^ = 0.53, *p* = 0.465
caterpillar	oecobiid spider	standard	1-day	12	*χ*^2^ = 1.20, *p* = 0.273	12	*χ*^2^ = 1.20, *p* = 0.273
caterpillar	oecobiid spider	spider-only	7-day	—	—	16	*χ*^2^ = 0.13, *p* = 0.715
caterpillar	oecobiid spider	spider-only	1-day	—	—	12	*χ*^2^ = 1.20, *p* = 0.273
aphid	oecobiid spider	standard	7-day	14	*χ*^2^ = 0.13, *p* = 0.715	15	*χ*^2^ = 0.00, *p* = 1
aphid	oecobiid spider	standard	1-day	16	*χ*^2^ = 0.13, *p* = 0.715	16	*χ*^2^ = 0.13, *p* = 0.715

^a^Number that chose prey 2 (*Anopheles* male) significantly more than number that chose prey 1 (blood *Culex* female).

**Table 7.** Preference strengths of *Evarcha culicivora* determined from complete series of simultaneous-presentation prey-choice experiments (table 6). Column 1: prey 1. Headings for each other column: prey 2. Strong preference for prey 1 (s): significantly more test spiders chose prey 1 than chose prey 2 after a 7-day fast. Weak preference for prey 1 (w): no significant choice after 7-day fast, but significantly more test spiders chose prey 1 than chose prey 2 after a 1-day pre-trial fast. Nil preference (n): no significant choice after 7-day and 1-day fasts. Strength of preference by adult test spiders indicated first, followed by strength of preference by juvenile test spiders. For details about prey, see table 3. Minus sign in front of w (2 instances, both with blood female *Culex* as prey 1): significantly more test spiders chose prey 2 after 1-day fast (contributes to preference index for prey 2 instead for prey 1: see table 10); otherwise s and w indicate preference for prey 1. For Nelson & Jackson [12] data, juveniles 2.0 mm in body length; 3.0 mm in all other instances.

	blood *Culex* female	no-blood *Anopheles* female	no-blood *Culex* female	*Anopheles* male	*Culex* male	ghost midge	chironomid midge	vinegar fly	mayfly	fruit fly	cricket	caterpillar	aphid	oecobiid spider
blood female *Anopheles*	w^a^, s^a^	s^a^, s^a^	s^a^, s^a^	s^a^, s^a^	s, s	s, s	s^b^, s^b^	s, s	s, s	s, s	s, s	s, s	s, s	s, s
blood *Culex* female	—	s^a^, -w^a^	s^a^, s^a^	s, -w	s^a^,s^a^	s, s	s^b^, s^b^	s, s	s, s	s, s	s, s	s, s	s, s	s, s
no-blood *Anopheles* female		—	w^a^, s^a^	w^a^, s^a^	w^a^, s^a^	w, s	w^b^, s^b^	s, s	s, s	s, s	s, s	s, s	s, s	s, s
no-blood *Culex* female			—	n^a^, n^a^	n^a^, n^a^	n, n	n^b^, n^b^	w, w	w, w	s, s	s, s	s, s	s, s	s, s
*Anopheles* male				—	n^a^, s^a^	n, w	n^b^, w^b^	w, w	w, w	s, s	s, s	s, s	s, s	s, s
*Culex* male					—	n, n	n^b^, n^b^	w, w	w, w	s, s	s, s	s, s	s, s	s, s
ghost midge						—	n, n	w, w	w, w	s, s	s, s	s, s	s, s	s, s
chironomid midge							—	w, w	w, w	s, s	s, s	s, s	s, s	s, s
vinegar fly								—	n, n	n, n	s, s	s, s	s, s	s, s
mayfly									—	n, n	w, w	s, s	s, s	s, s
fruit fly										—	s, w	s, s	s, s	s, s
cricket											—	n, n	n, n	n, n
caterpillar												—	n, n	n, n
aphid													—	n, n

^a^From Nelson & Jackson [12]. s in this table corresponds to medium in table 2.

^b^Same for another chironomid midge, *Clinotanypus claripennis*, in Nelson & Jackson [12].

Findings from the complete series were more complex when other mosquitoes were paired with non-mosquito prey (tables 6 and 7), where ‘other mosquitoes’ refers to no-blood *Culex* females, *Culex* males and *Anopheles* males. Adults and juveniles expressed no preference when no-blood *Culex* females or *Culex* males were paired with midges (tables 6 and 7). However, there was a juvenile--adult difference when *Anopheles* males were paired with midges: adult test spiders expressed no preference for the *Anopheles* male but juveniles expressed a weak preference for the *Anopheles* male. Juveniles and adults expressed weak preferences for no-blood *Culex* females, *Culex* males and *Anopheles* males when the alternatives were vinegar flies or mayflies, and they expressed strong preferences when the alternatives were fruit flies, crickets, caterpillars, aphids or oecobiid spiders.

In the mosquito series (table 8), we paired *Anopheles* blood or no-blood female mosquitoes with 15 non-mosquito prey species that had not been used in the complete series (table 7) or in any previously published experiments (table 1). For this series, we found that the number of adult and juvenile test spiders that chose the mosquito was always significantly larger than the number that chose the non-mosquito prey. As the pre-trial fast duration was always 7 days, our findings from the mosquito series corresponded to the definition that we used in the complete series for ‘strong preference’.

**Table 8.** Findings for adult females and juveniles of *Evarcha culicivora* (‘test spiders’) in mosquito series of simultaneous-presentation prey-choice experiments. For each experiment (row), 25 test spiders chose one of the two prey (i.e. *n* = 25 for each row). Blood: blood *Anopheles* female. No-blood: no-blood *Anopheles* female. See table 3 for details pertaining to prey. For all experiments, there was a pre-trial fast of 7 days. Data analysis: tests of goodness of fit (null hypothesis: as likely to choose prey 2 as to choose prey 1).

		adult female test spiders		juvenile test spiders	
prey 1	prey 2	chose prey 1	test of goodness of fit	chose prey 1	test of goodness of fit
blood	assassin bug	24	*χ*^2^ = 21.16, *p *< 0.001	25	*χ*^2^ = 25.00, *p *< 0.001
blood	barklouse	20	*χ*^2^ = 9.00, *p* = 0.003	20	*χ*^2^ = 9.00, *p* = 0.003
blood	brown rice hopper	25	*χ*^2^ = 25.00, *p *< 0.001	22	*χ*^2^ = 14.44, *p *< 0.001
blood	clubionid spider	25	*χ*^2^ = 25.00, *p *< 0.001	24	*χ*^2^ = 21.16, *p *< 0.001
blood	cockroach	23	*χ*^2^ = 17.64, *p *< 0.001	20	*χ*^2^ = 9.00, *p* = 0.003
blood	green leaf hopper	22	*χ*^2^ = 14.44, *p *< 0.001	24	*χ*^2^ = 21.16, *p *< 0.001
blood	hersiliid spider	25	*χ*^2^ = 25.00, *p *< 0.001	25	*χ*^2^ = 25.00, *p *< 0.001
blood	house fly	22	*χ*^2^ = 14.44, *p *< 0.001	23	*χ*^2^ = 17.64, *p *< 0.001
blood	jumping spider	23	*χ*^2^ = 17.64, *p *< 0.001	25	*χ*^2^ = 25.00, *p *< 0.001
blood	long-legged fly	21	*χ*^2^ = 11.56, *p *< 0.001	21	*χ*^2^ = 11.56, *p *< 0.001
blood	mantis	25	*χ*^2^ = 25.00, *p *< 0.001	24	*χ*^2^ = 21.16, *p *< 0.001
blood	moth fly	23	*χ*^2^ = 17.64, *p *< 0.001	23	*χ*^2^ = 17.64, *p *< 0.001
blood	nephilid spider	23	*χ*^2^ = 17.64, *p *< 0.001	25	*χ*^2^ = 25.00, *p *< 0.001
blood	whitefly	19	*χ*^2^ = 6.76, *p* = 0.009	25	*χ*^2^ = 25.00, *p *< 0.001
blood	wolf spider	22	*χ*^2^ = 14.44, *p *< 0.001	24	*χ*^2^ = 21.16, *p *< 0.001
no-blood	assassin bug	24	*χ*^2^ = 21.16, *p *< 0.001	22	*χ*^2^ = 14.44, *p *< 0.001
no-blood	barklouse	20	*χ*^2^ = 9.00, *p* = 0.003	23	*χ*^2^ = 17.64, *p *< 0.001
no-blood	brown rice hopper	19	*χ*^2^ = 6.76, *p* = 0.009	21	*χ*^2^ = 11.56, *p *< 0.001
no-blood	clubionid spider	24	*χ*^2^ = 21.16, *p *< 0.001	25	*χ*^2^ = 25.00, *p *< 0.001
no-blood	cockroach	25	*χ*^2^ = 25.00, *p *< 0.001	25	*χ*^2^ = 25.00, *p *< 0.001
no-blood	green leaf hopper	21	*χ*^2^ = 11.56, *p *< 0.001	22	*χ*^2^ = 14.44, *p *< 0.001
no-blood	hersiliid spider	21	*χ*^2^ = 11.56, *p *< 0.001	24	*χ*^2^ = 21.16, *p *< 0.001
no-blood	house fly	23	*χ*^2^ = 17.64, *p *< 0.001	23	*χ*^2 ^= 17.64, *p *< 0.001
no-blood	jumping spider	23	*χ*^2 ^= 17.64, *p *< 0.001	25	*χ*^2 ^= 25.00, *p *< 0.001
no-blood	long-legged fly	20	*χ*^2 ^= 9.00, *p* = 0.003	21	*χ*^2 ^= 11.56, *p *< 0.001
no-blood	mantis	23	*χ*^2 ^= 17.64, *p *< 0.001	24	*χ*^2 ^= 21.16, *p *< 0.001
no-blood	moth fly	24	*χ*^2 ^= 21.16, *p *< 0.001	21	*χ*^2 ^= 11.56, *p *< 0.001
no-blood	nephilid spider	22	*χ*^2 ^= 14.44, *p *< 0.001	24	*χ*^2 ^= 21.16, *p *< 0.001
no-blood	whitefly	20	*χ*^2 ^= 9.00, *p* = 0.003	21	*χ*^2 ^= 11.56, *p *< 0.001
no-blood	wolf spider	21	*χ*^2 ^= 11.56, *p *< 0.001	25	*χ*^2 ^= 25.00, *p *< 0.001

### 3.3. Choice between non-mosquito species

Adult and juvenile test spiders expressed no preference when different midge species were paired with each other in the complete series (tables 6 and 7) and in the non-mosquito series (table 9). However, adult and juvenile test spiders expressed a weak preference in the complete series for midges when the alternative was a vinegar fly or mayfly and a strong preference for midges when the alternative was a fruit fly, cricket, caterpillar, aphid or oecobiid spider.

**Table 9.** Findings for *Evarcha culicivora* (‘test spiders’) in the non-mosquito series of prey-choice experiments (see the text). For each experiment (row), simultaneous-presentation testing was used and 25 spiders chose one of the two prey (i.e. *n* = 25 for each row). For details concerning prey, see table 3. For all experiments, there was a pre-trial fast of 1 day. Data analysis: tests of goodness of fit (null hypothesis: as likely to choose prey 2 as to choose prey 1).

		adult female test spiders		juvenile test spiders	
prey 1	prey 2	chose prey 1	test of goodness of fit	chose prey 1	test of goodness of fit
*Chaoborus* sp.	*Ablabesmyia nilotica*	11	*χ*^2 ^= 0.36, *p* = 0.549	10	*χ*^2 ^= 1.00, *p* = 0.317
*Chaoborus* sp.	*Chironomus imicola*	14	*χ*^2 ^= 0.36, *p* = 0.549	12	*χ*^2 ^= 0.04, *p* = 0.841
*Chaoborus* sp.	*Clinotanypus claripennis*	11	*χ*^2 ^= 0.36, *p* = 0.549	13	*χ*^2 ^= 0.04, *p* = 0.841
*Chaoborus* sp.	*Conochironomus acutistus*	14	*χ*^2 ^= 0.36, *p* = 0.549	14	*χ*^2 ^= 0.36, *p* = 0.549
*Nilodorum brevibucca*	*Ablabesmyia nilotica*	15	*χ*^2 ^= 1.00, *p* = 0.317	15	*χ*^2 ^= 1.00, *p* = 0.317
*Nilodorum brevibucca*	*Chironomus imicola*	10	*χ*^2 ^= 1.00, *p* = 0.317	9	*χ*^2 ^= 1.96, *p* = 0.162
*Nilodorum brevibucca*	*Clinotanypus claripennis*	15	*χ*^2 ^= 1.00, *p* = 0.317	14	*χ*^2 ^= 0.36, *p* = 0.549
*Nilodorum brevibucca*	*Conochironomus acutistus*	16	*χ*^2 ^= 1.96, *p* = 0.162	13	*χ*^2 ^= 0.04, *p* = 0.841
*Ablabesmyia nilotica*	*Chironomus imicola*	12	*χ*^2 ^= 0.04, *p* = 0.841	14	*χ*^2 ^= 0.36, *p* = 0.549
*Ablabesmyia nilotica*	*Clinotanypus claripennis*	12	*χ*^2 ^= 0.04, *p* = 0.841	8	*χ*^2 ^= 3.24, *p* = 0.072
*Ablabesmyia nilotica*	*Conochironomus acutistus*	14	*χ*^2 ^= 0.36, *p* = 0.549	11	*χ*^2 ^= 0.36, *p* = 0.549
*Chironomus imicola*	*Clinotanypus claripennis*	12	*χ*^2 ^= 0.04, *p* = 0.841	9	*χ*^2 ^= 1.96, *p* = 0.162
*Chironomus imicola*	*Conochironomus acutistus*	9	*χ*^2 ^= 1.96, *p* = 0.162	11	*χ*^2 ^= 0.36, *p* = 0.549
*Clinotanypus claripennis*	*Conochironomus acutistus*	11	*χ*^2 ^= 0.36, *p* = 0.549	13	*χ*^2 ^= 0.04, *p* = 0.841
oecobiid spider	wolf spider	13	*χ*^2 ^= 0.04, *p* = 0.841	12	*χ*^2 ^= 0.04, *p* = 0.841
oecobiid spider	jumping spider	14	*χ*^2 ^= 0.36, *p* = 0.549	9	*χ*^2 ^= 1.96, *p* = 0.162
oecobiid spider	nephilid spider	16	*χ*^2 ^= 1.96, *p* = 0.162	13	*χ*^2 ^= 0.04, *p* = 0.841
wolf spider	nephilid spider	10	*χ*^2 ^= 1.00, *p* = 0.317	9	*χ*^2 ^= 1.96, *p* = 0.162
wolf spider	jumping spider	13	*χ*^2 ^= 0.04, *p* = 0.841	13	*χ*^2 ^= 0.04, *p* = 0.841
jumping spider	nephilid spider	15	*χ*^2 ^= 1.00, *p* = 0.317	12	*χ*^2 ^= 0.04, *p* = 0.841

Adult and juvenile test spiders in the complete series (tables 6 and 7) expressed no preference when vinegar flies were paired with mayflies or fruit flies but they expressed a strong preference for vinegar flies when paired with crickets, caterpillars, aphids or oecobiid spiders. Adult and juvenile test spiders also expressed no preference when mayflies were paired with fruit flies, but they expressed a weak preference for mayflies when the alternative was a cricket and they expressed a strong preference for mayflies when the alternative was a caterpillar, aphid or oecobiid spider. Adult and juvenile test spiders also expressed a strong preference for fruit flies when the alternative was a caterpillar, aphid or oecobiid spider; however, adults expressed a strong preference, and juveniles only a weak preference, for fruit flies when the alternative was a cricket. When crickets, caterpillars, aphids and oecobiid spiders were paired with each other, no preferences were expressed by adult or juvenile test spiders. Moreover, in the non-mosquito series, adult and juvenile test spiders expressed no preferences when the different spider species were paired with each other (table 9).

### 3.4. Effect of rearing diet on prey-choice behaviour

When juvenile test spiders in the complete series were reared on a spider-only diet (table 6), their preferences were never significantly different (*χ*^2^ tests of independence) from the preferences of juvenile spiders that had been on the standard diet: blood *Anopheles* females paired with ghost midges (*χ*^2^ = 1.02, *p* = 0.313), blood *Anopheles* females paired with oecobiid spiders (*χ*^2^ = 2.07, *p* = 0.150), no-blood *Anopheles* females paired with ghost midges (*χ*^2^ = 0.13, *p* = 0.718), no-blood *Anopheles* females paired with oecobiid spiders (*χ*^2^ = 2.96, *p* = 0.085), *Anopheles* males paired with ghost midges (7-day fast: *χ*^2^ = 0.27, *p* = 0.605; 1-day fast: *χ*^2^ = 0.22, *p* = 0.640), *Anopheles* males paired with oecobiid spiders (*χ*^2^ = 1.07, *p* = 0.301), ghost midges paired with oecobiid spiders (*χ*^2^ = 0.16, *p* = 0.688), vinegar flies paired with oecobiid spiders (*χ*^2^ = 4.04, *p* = 0.044) or caterpillars paired with oecobiid spiders (7-day fast: *χ*^2^ = 0.07, *p* = 0.795; 1-day fast: *χ*^2^ = 0, *p* = 1).

### 3.5. Preference indexes

Using our new data from the complete series (table 7) combined with the data in Nelson & Jackson [12] from pairing mosquito types (table 2), we calculated a preference index (table 10) for each of the 15 prey types from the complete series. Our new data include blood *Anopheles* females paired with *Culex* males and blood *Culex* females paired with *Anopheles* males (table 6), these being the cells missing from Nelson& Jackson [12]. The resulting indexes ranged from 0 for prey that was never preferred to another prey and 28 for a prey that was strongly preferred to all other prey.

**Table 10.** Preference indexes for 15 prey categories used in complete series of simultaneous-presentation prey-choice experiments (see the text and table 7). For each pairing with another prey type, each prey category given a score of 0 when no preference was expressed (not significant after 7-day and 1-day prey-trial fast), 1 when only a weak preference was expressed (significantly more test spiders chose this prey type after a 1-day, but not 7-day, fast) and 2 when a strong preference was expressed (significantly more chose this prey type after 7-day fast). Preference index for prey category: sum of scores for that prey category paired with each other category.

preference index	juvenile test spider	adult test spider
28	blood *Anopheles* female	—
27	—	blood *Anopheles* female
26	—	blood *Culex* female
25	no-blood *Anopheles* female	—
24	—	—
23	—	—
22	blood *Culex*	—
21	—	—
20	—	—
19	—	no-blood *Anopheles* female
18	—	—
17	*Anopheles* male	—
16	—	—
15	—	—
14	—	—
13	—	—
12	no-blood *Culex* female, *Culex* male, ghost midge and chironomid midge	no-blood *Culex* female, *Anopheles* male, *Culex* male, ghost midge and chironomid midge
11	—	—
10	—	—
9	—	—
8	vinegar fly	vinegar and fruit fly
7	mayfly and fruit fly	mayfly
6	—	—
5	—	—
4	—	—
3	—	—
2	—	—
1	—	—
0	cricket, caterpillar, aphid, spider	cricket, caterpillar, aphid, spider

Irrespective of whether test spiders were juveniles or adults, and irrespective of diet (table 10), the preference indexes for crickets, caterpillars, aphids and oecobiid spiders were 0, the indexes for vinegar flies, mayflies and fruit flies were clustered at 7 and 8, and the indexes for no-blood *Culex* females, *Culex* males and midges were clustered at 12. The highest preference indexes were for blood *Anopheles* females (27 for juveniles and 28 for adults).

There were, however, distinct juvenile–adult differences when the prey was a no-blood *Anopheles* female, an *Anopheles* male or a blood *Culex* female. The preference index for *Anopheles* males was 17 when test spiders were juveniles but only 12 when test spiders were adults. The preference index for no-blood *Anopheles* females was 25 when test spiders were juveniles, but only 19 when test spiders were adults. The preference index for blood *Culex* females was 26 when test spiders were adults, but only 22 when test spiders were juveniles.

## 4. Discussion

### 4.1. *Evarcha culicivora*'s natural diet

Determining a spider's natural diet can be a daunting task, but perhaps less so when the spider lives in a web because identifiable prey can often be found in the web, with some web-building spiders wrapping their prey in silk and then leaving it in the web as a larder to feed from at a later time [24–27] (for a cautionary note, see [28]). Salticids are less accommodating because, barring a few exceptions [29], these are predators that find, capture and eat their prey without using a web [30]. In the field, prey can be collected and identified whenever a salticid is encountered in the act of feeding, but determining natural diet in this way is a slow, laborious process. It is also likely to underestimate the number of very small or very masticated prey being eaten by spiders, as these would be less likely to be noted opportunistically.

Understandably, the sample sizes in salticid prey records are often too small for robust conclusions about natural diet, but it can be hard to say how small is too small. However, even when we made an admittedly arbitrary decision to accept 20 clearly specified prey records as a minimum, we found only a few published records for salticids other than *E*. *culicivora* (table 11). The prey records for *E*. *culicivora* (1115) are more than 10 times larger than records for any of these other salticid species, but records for *E*. *culicivora* were accumulated over a span of 19 years in the course of doing intensive research on this particular species. Moreover, this species is from a field site almost on the equator where all life-cycle stages are active and abundant year round. For most salticid species, it would seem unrealistic to expect prey records of comparable size.

**Table 11.** Sample sizes in studies on the prey of salticids in the field.

salticid species	no. records of prey	source
*Aelurillus m-nigrum*	58	[31]
*Aelurillus muganicus*	64	[32]
*Cyrba algerina*	59	[33]
*Heliophanus dunni*	50	[34]
*Jacksonoides queenslandicus*	25	[35]
*Menemerus semilimbatus*	96	[36]
*Menemerus taeniatus*	62	[37]
*Mexcala elegans*	64	[38]
*Phidippus johnsoni*	33	[39]
*Phidippus audax*	21	[40]
*Portia fimbriata*	24	[41]
*Portia fimbriata*	61	[42]
*Paracyrba wanlessi*	84	[43]
*Salticus tricinctus*	40	[44]
*Salticus austinensis*	46	[45]
*Siler cupreus*	24	[46]
*Tauala lepidus*	22	[47]

From our prey records, we can safely conclude that *E*. *culicivora* lives up to its species’ name. It certainly eats mosquitoes. It also appears to have a narrow natural diet which suggests that, for this predator, ‘stenophagy’ is an appropriate expression. However, it might be prudent to consider what we achieve and what we imply when we characterize *E*. *culicivora*'s natural diet in this way.

The taxonomic resolution we adopt will influence our use of the terms ‘monophagy’, ‘stenophagy’ and ‘euryphagy’. For example, if we use phyla as our level of taxonomic resolution, then we would conclude that *E*. *culicivora*'s natural diet is an instance of monophagy because we would then say this spider eats a single type of prey, in this case arthropods. However, if we use orders or families as our level of taxonomic resolution, then characterizing *E*. *culicivora*'s natural diet as being a distinctive example of stenophagy becomes more interesting. Most of the prey on which we found *E*. *culicivora* feeding came from one insect order (Diptera) and most of these came from a single dipteran family, the Culicidae (i.e. mosquitoes). A natural diet biased strongly toward a single insect family may be unusual for spiders or for predators in general, but this is conjecture, not simply a fact. Moreover, it is important to appreciate that stenophagy is not a synonym for specialization and natural diet is conceptually distinct from preference [10].

### 4.2. Alignment between *Evarcha culicivora*'s natural diet and preferences

Although preference cannot be determined on the basis of natural diet alone, we found striking instances of alignment between the two for *E*. *culicivora*. In particular, female mosquitoes dominated the prey records from the field and, in prey-choice experiments, the highest preference indexes were for female mosquitoes (tables 5 and 7). Male mosquitoes and midges were the next most prevalent prey types in the prey records and, in experiments, the preference indexes for these prey types were higher than the preference indexes for non-mosquito or non-midge prey (table 10).

All non-mosquito and non-midge prey types were scarce (less than 4%) in our field records (table 5). Data on prey choice from the complete series revealed no evidence of crickets, caterpillars, aphids or oecobiid spiders being preferred to any other prey. As there was also no expression of preference in the non-mosquito series when different spider species were paired with each other (table 9), we propose that *E*. *culicivora* expresses no preference for spiders in general. However, there were instances of preference being expressed for fruit flies and for vinegar flies, these being prey we chose as proxies for ‘other dipterans’ in the complete series. There were also instances in the complete series of *E*. *culicivora* expressing preference for mayflies. Yet ‘other dipterans’ and mayflies were scarce in the field records.

In our non-mosquito series of prey-choice experiments (table 9), as well as in the complete series (table 7), there was no evidence to suggest that *E*. *culicivora* distinguishes between different kinds of midges, but there was evidence that *E*. *culicivora* distinguishes between different kinds of mosquitoes. Previous research has revealed that *E*. *culicivora* has a preference for anophelines [12,15] when the alternatives are culicines, as well as for blood female mosquitoes when the alternatives are no-blood female mosquitoes [11–13]. However, anophelines were not markedly more common than culicines in our field prey records. Moreover, blood females were less, not more, common than no-blood female mosquitoes in our prey records (table 5).

These apparent misalignments (i.e. instances of ‘preference’ not being in close alignment to natural diet) do not surprise us because the definition of ‘preference’ does not somehow demand alignment. However, the extent to which *E*. *culicivora*'s preferences for particular mosquitoes really are misaligned with prey eaten in the field is uncertain because we were limited in our capacity to identify prey that we removed from feeding individuals, especially when the prey was a soft-bodied mosquito that the spider had already begun to crush with its chelicerae. This meant that often we could not determine whether a mosquito was an anopheline or a culicine (table 5) and, owing to the predator actively digesting blood that might have been present, our methods (i.e. simply pressing on the mosquito's abdomen and recording whether there were signs of liquid blood) may have seriously underestimated the numbers of female mosquitoes that had been carrying blood when captured.

### 4.3. Prey categorization by *Evarcha culicivora*

In the complete series, we used 15 prey types chosen on the basis of findings from earlier prey-choice experiments (table 1) as well as on the basis of our records of *E*. *culicivora*'s prey in the field. Besides using these data as evidence of preferences, we can use these data for proposing the types of prey *E*. *culicivora* characterized as being members of different visual categories. The rationale for these hypotheses is that the expression of preference is evidence *E*. *culicivora* has the capacity and motivation to discriminate by sight between the two types of prey being considered in an experiment. In all instances, we simply looked for instances of a preference, leaving aside the distinction between strong and weak preference. When test spiders failed to choose one prey type significantly more often than another, we had no comparable evidence of capacity and motivation and we recorded experimental outcomes as instances of ‘nil preference’.

The categories were internally consistent, meaning that, whenever a preference was expressed for a member of a particular category and this member was paired with any member of any other category, the direction of preference (i.e. which of the two prey types was chosen significantly more often) was consistent in each instance. Moreover, there was no evidence of a preference whenever a member of one category was paired with another member of the same category.

Using this procedure, we identified seven categories for juveniles and six for adults (table 12). Blood *Anopheles* females, blood *Culex* females and no-blood *Anopheles* females were three distinct categories for adults and for juveniles. *Anopheles* males were also a distinct category for juveniles, but for adults *Anopheles* males were members of a multi-type group that also included midges and *Culex* males. There were another three multi-type groups that juveniles and adult females responded to as three distinct categories and these were the categories with the lowest preference indexes (tables 10 and 12).

**Table 12.** Prey categories for *Evarcha culicivora* juveniles and adult *E*. *culicivora* females, determined from complete series of simultaneous-presentation prey-choice experiments. See the text for category-derivation procedure and table 3 for details pertaining to prey. When applicable, preference index listed for each category (table 10). Each category given a letter code followed by listing of constituent prey types. Categories a, b, c, f and g applicable to juvenile and adult female test spiders. For juveniles, but not for adult females, d and e are distinct categories. Category de for adult females is inclusive of prey in categories d and e of juveniles. n.a.: not applicable.

prey category	juvenile preference index	adult female preference index
a. blood *Anopheles*	28	27
b. blood *Culex* female	22	26
c. no-blood *Anopheles* female	25	19
d. *Anopheles* male (17)	17	n.a.
e. no-blood *Culex* female, *Culex* male, ghost midge, chironomid midge (12)	12	n.a.
de. *Anopheles* male, no-blood *Culex* female, *Culex* male, ghost midge, chironomid midge (12)	n.a.	12
f. vinegar fly, fruit fly, mayfly (7–8)	7–8	7–8
g. cricket, caterpillar, aphid, spider (0)	0	0

The categories we determined from the complete series were remarkably coherent. Moreover, we discerned these categories regardless of whether test spiders had been on a standard diet or on a ‘spider-only’ rearing diet. On this basis, we can characterize these categories as being ‘innate’, but ‘innate’ does not mean ‘inflexible’. Although beyond the scope of our present study, we would expect operant conditioning and other environmental shaping to influence prey categorization by *E*. *culicivora*. Furthermore, we have not addressed questions about the origins and adaptive significance of the way *E.culicivora* categorizes prey, as this was also beyond the scope of our study.

### 4.4. *Evarcha culicivora's* own prey-classification system

When categorizing a predator's prey, it may be tempting to rely on formal scientific taxonomy, which is appropriate when considering food webs and other topics in community ecology [48–51]. It is also common practice to use scientific taxonomy when sampling the relative availability of different kinds of prey in a predator's habitat and when comparing these samples with records of the prey actually eaten by the predator. When found, significant disparities indicate that the predator's natural diet is biased toward a subset of available potential prey, and ‘ecological selectivity’ is a convenient expression for these disparities.

Although determining ecological selectivity was not an objective in our study, we are confident that sampling for potential prey in *E*. *culicivora*'s habitat would reveal that midges, known locally as ‘lake flies’, vastly outnumber mosquitoes along the shores of Lake Victoria [52,53]. Yet it is important to emphasize that ecological selectivity is not our basis for concluding that *E*. *culicivora* has a strong preference for mosquitoes as prey. Our primary goal has been to achieve a better understanding of predatory specialization and, for this, we need an understanding of the different ways in which a predator experiences and classifies its prey [1,10].

Unlike scientists, non-human predators do not rely on Latin names from formal taxonomy when they classify their prey. It is the predator's own prey-classification system, and not evidence from ecological selectivity, which highlights one of the most interesting discoveries about *E*. *culicivora*'s categorization of prey. This is a predator that does a lot of classifying. The insects that scientists assign to the family Culicidae are experienced by *E*. *culicivora* as more than a single prey category, with the distinctions that matter to *E*. *culicivora* including whether the mosquito is an anopheline or a culicine, whether it is a male or a female and whether it is a female that is or is not carrying blood. Moreover, the juveniles of *E*. *culicivora* adopt an *Anopheles*-specific prey-capture method [54] (see also [1]) and stronger preferences are expressed by juveniles than by adults for anophelines [12] (table 12).

*Paracyrba wanlessi* is another salticid that specializes at preying on mosquitoes, but it does not classify its prey in the same way as *E*. *culicivora* [1]. For *P*. *wanlessi*, the distinctions that matter are whether a mosquito is an adult or juvenile and whether the prey is in or away from water [55], but there is no evidence of these categories being relevant to *E*. *culicivora*. Although *E*. *culicivora* and *P*. *wanlessi* can both be said to be salticid species that ‘prefer mosquitoes’ as prey, this simplistic statement hides major differences in how these two predators classify their prey.

Furthermore, in prey-choice experiments using another 19 salticid species from East Africa, no evidence was found of any of these salticids discriminating between blood meals (blood-carrying *An*. *gambiae s*.*s*. females) and non-blood meals (lake flies or *An*. *gambiae s*.*s*. males) [13]. Yet, we need to be open to the logical possibility of finding pronounced ecological selectivity toward mosquitoes by predators that express no preference for mosquitoes. Many insectivorous predators, including many salticids, may experience mosquitoes not as a distinct prey category and instead as just another ‘bug’ (see [8,56,57]). It is at least a logical possibility that some of these predators eat disproportionately more anopheline than culicine mosquitoes, female than male mosquitoes or blood-carrying than bloodless mosquitoes, without any of these being distinct categories within a prey-classification system adopted by these predators.

## Data accessibility

All data are included in tables 4–6, 8 and 9.
